# Competencies and Needs of Older Volunteers: A Comparative Survey

**DOI:** 10.1097/jnr.0000000000000712

**Published:** 2025-11-26

**Authors:** Li-Ching YANG, Ching-Yi LAI, Kuei-Min CHEN, Meng-Chin CHEN, Chiang-Ching CHANG, Tzu-Yu LIN, Chuan-Feng KUO

**Affiliations:** 1 Department of Nursing, Fooyin University, Kaohsiung, Taiwan; 2 College of Nursing, Kaohsiung Medical University, Kaohsiung, Taiwan; 3 Master Program of Long-term Care in Aging, Kaohsiung Medical University, Kaohsiung, Taiwan; 4 Center for Long-term Care Research, Kaohsiung Medical University, Kaohsiung, Taiwan; 5 Department of Medical Research, Kaohsiung Medical University Hospital, Kaohsiung, Taiwan; 6 Department of Nursing, Yuhing Junior College of Health Care and Management, Kaohsiung, Taiwan; 7 Department of Medical Sociology and Social Work, Kaohsiung Medical University, Kaohsiung, Taiwan; † Contributed equally

**Keywords:** community care center, competencies and needs, older volunteer, primary preventive care

## Abstract

**Background:**

Considering Taiwan’s impending status as a super-aged society, active involvement in volunteer work has been identified as a means to facilitate active aging, enhance social participation, and sustain the mental and physical well-being of older adults. However, volunteers require proper training to cope with the complex situations they encounter as first-line care providers. Existing volunteer training programs lack pretraining assessments to gauge preparedness for the challenges faced by frontline care providers.

**Purpose:**

This study was designed to identify the demographic characteristics, competencies, and needs of older adult volunteers and investigate the influence of demographic variables on key caregiving competencies.

**Methods:**

A comparative cross-sectional survey design was adopted, and the data were collected using a structured self-reporting questionnaire. Simple random sampling was employed to select 73 community care centers in a southern city in Taiwan, with 1,000 older adult volunteer caregivers enrolled as participants.

**Results:**

The average age of the participants was 71.79 years, and most were female (80.8%), were married (64.2%), had a senior high school or higher level of education (51.9%), and had over 5 years of volunteer work experience (68.0%). The mean score for overall competencies and needs of older adults, as measured using the Older Volunteer Competency Scale, was 3.91±2.66. The three dimensions of this scale, ranked in descending mean score order, were volunteer service skills (4.49±2.76), volunteer service awareness (3.91±2.85), and volunteer interpersonal interaction (3.54±2.86). Age, volunteering experience, and educational level were shown to significantly influence the level of need. In terms of overall competencies and needs, the oldest age group had a higher needs level than the middle-aged group. Also, those participants with the least volunteering experience and the lowest educational level groups had the highest related needs. In terms of service awareness, both the youngest and oldest age groups demonstrated greater needs than the middle-aged group. Similarly, those with an elementary school or junior high school level of education were identified as having greater needs than those with a senior high school or higher level of education. In terms of service skills, those participants with the least volunteering experience and the lowest educational level groups had the greatest related needs. In terms of interpersonal interaction, those participants in the least volunteering experience and lowest educational level groups had the greatest related needs.

**Conclusions/Implications for Practice:**

The needs of older volunteers are significantly influenced by age, length of volunteering experience, and educational level. Community care centers should conduct pre-employment assessments to optimally allocate tasks as well as provide opportunities for on-the-job training.

## Introduction

According to the United Nations (2022), the proportion of individuals aged ≥65 years is expected to increase from 10% of the global population in 2022 to 16% by 2050. This demographic shift, coupled with declining birth rates, has accelerated population aging. Consequently, active aging, which promotes empowering older adults to enhance their social participation, secure employment opportunities, achieve financial stability, and foster independent, autonomous, and safe living conditions, has garnered global attention (Hsu et al., 2019; Li & Hsieh, 2020). The concept of active aging aligns with that of primary preventive care, which advocates enhancing the health of community-dwelling older adults by strengthening social support for older adults, elevating their health awareness, and promoting health-conscious behavior with the overarching goal of enabling them to lead healthy lives and avoid/delay the onset of frailty, disability, and disease (Chiu et al., 2020; Yang et al., 2019). Within these frameworks, the promotion of social participation is regarded as a means for older adults to access social support, improve their health, and extend overall longevity.

The involvement of older adults in volunteer services is a pivotal strategy and indicator of successful active aging (Hsu et al., 2019; Li & Hsieh, 2020). Engaging in volunteer services, which involves sustained and active social participation, enables older adults to discover the meaning of life; gain confidence, self-esteem, and autonomy; maintain health-promoting behaviors and habits; enhance overall sense of well-being, satisfaction, and quality of life (Huang, 2019; Kleiner et al., 2022); alleviate symptoms of depression and risk of death; promote healthy aging and social well-being; and challenge stereotypical and biased views toward older adults (Filges et al., 2020; Huang, 2019; Huo et al., 2021). Therefore, encouraging older adults to actively participate in volunteer services has emerged as a new trend and strategy (Huang, 2019; Ministry of Health and Welfare, Taiwan, ROC, 2021a). In Australia, older adults account for 30% of all volunteers (Volunteering Australia, 2021), while in the United States, 57.2% of all volunteers are older adults (AmeriCorps, 2021), making them the backbone of that country’s volunteer labor pool.

In 2023, the population of individuals aged 65 and older reached 4.3 million, accounting for 18.35% of the total population (Ministry of the Interior, Taiwan, ROC, 2024). This percentage is projected to increase to 20% by 2025 and 28% by 2036 (National Development Council, Taiwan, ROC, 2024; Ministry of Health and Welfare, Taiwan, ROC, 2021a), indicating a pace of population aging twice as rapid as that observed in Western countries. In response to this trend, Taiwan initiated a program in 2005 to establish community care centers, aiming to promote active aging and reinforce the provision of primary preventive care to community-dwelling older adults (Hsieh, 2018; Yeh, 2021). Primary preventive care services offered by these community care centers include home visits, greeting phone calls, meal delivery, consultations, referrals, and health-promoting activities. This initiative was followed up by the Long-term Care Plan 2.0 program in 2016, which introduced community-based long-term care stations that transformed the community care center model. Community long-term care stations retain the services of community care centers while introducing additional activities designed to promote social participation and prevent or delay disability (Hsieh, 2018; Ministry of Health and Welfare, Taiwan, ROC, 2016). As Taiwan’s population aging policies encourage middle-aged and older individuals to engage in volunteer services to promote active aging (Hsu et al., 2019; Ministry of Health and Welfare, Taiwan, ROC, 2021a), older adults now account for roughly 30.4% of all volunteers in the country (Ministry of Health and Welfare, Taiwan, ROC, 2021b), making them the primary workforce providing diversified primary preventive care services.

Older volunteers, generally defined as individuals aged 65 or above, participate in community-based services such as home visits, greeting phone calls, meal delivery, consultations, referrals, and health-promoting activities (Fu et al., 2022; Ministry of Health and Welfare, Taiwan, ROC, 2016; Yeh, 2021). The continuous acquisition of new knowledge and skills has been identified as a factor that positively influences the perceived value of volunteer services, attitudes toward participating in volunteer services, and intention to sustain engagement in volunteer services (Fu et al., 2022; Jongenelis et al., 2020; Same et al., 2020). In Taiwan, volunteer training is categorized into either preservice training, which is organized and supervised by the government and is not mandatory, or in-service training, which is self-organized by the organizations in which the volunteers serve (Fu et al., 2022; Ministry of Health and Welfare, Taiwan, ROC, 2018). Notably, the majority (87.3%) of volunteer training programs for older adults are in the preservice training category, with only 24.9% categorized as in-service training. Moreover, a significant 38.8% of older adult volunteers have never received any form of training (Ministry of Health and Welfare, Taiwan, ROC, 2022). Preservice training plays a crucial role in helping older volunteers identify with, and gain the basic knowledge and mental preparedness necessary to engaging in volunteer services. However, in-service training is equally vital to assist them in acquiring ongoing support and unlocking opportunities for personal growth (Ministry of Health and Welfare, Taiwan, ROC, 2018).

The majority of community care center volunteers have expressed the need for diverse in-service training programs to strengthen their professional skills. Nearly half (47%) have advocated for community centers to enhance their capacity to design and lead both dynamic and static health-promoting activities. Moreover, 54.1% have emphasized the need for community care centers to strengthen the implementation of health-promoting activities in the future, while approximately 40% have highlighted the importance of improving psychological support service-related competencies. Importantly, 46.6% of community-dwelling older adults have identified health-promoting activities as a key area in need of future service enhancement (Fu et al., 2022).

The literature on volunteerism generally acknowledges that the competencies required for volunteers in primary preventive care services fall into one of the following three key domains: (a) knowledge: understanding the role of volunteers; the physiological, psychological, and the spiritual changes associated with aging; and the principles of health promotion; (b) skills: proficiency in community resource linkage and referral and in designing and leading activities; and (c) interpersonal interaction: demonstrating problem-solving abilities, effective communication, and strong interpersonal skills (Fu et al., 2022; Kritz et al., 2021; Kung et al., 2023). Thus, having a comprehensive assessment tool that can evaluate these multiple dimensions is crucial for accurately analyzing volunteer competencies and identifying their needs. Furthermore, training programs for older volunteers must be planned in accordance with their competencies and needs (Association of Talent Development, 2019; Yeh, 2021) and consider their mental and social traits, development, and needs (Wei & Liang, 2017). The benefits include maximizing the relevance and effectiveness of training in the diverse knowledge and skills required to deliver primary preventive care and providing to older volunteers the integrated knowledge, skills, attitudes, and values critical to coping effectively with complex service scenarios (Mahmud et al., 2019; Mazhisham et al., 2018; Ministry of Health and Welfare, Taiwan, ROC, 2018).

For older adults, engaging in volunteer services promotes active aging, primary preventive care, self-support, mutual support, and lifelong learning. Training initiatives are pivotal to keeping volunteers motivated and enhancing intention to stay, making these a key strategy for the sustainable management of organizations (Fu et al., 2022; Kritz et al., 2021). Studies to date on older volunteers have focused primarily on organizational management, motivation for continuous participation, and positive effects (Jiang et al., 2021; Jongenelis et al., 2020; Kritz et al., 2021), with limited exploration of the competencies and needs of older volunteers in terms of providing primary preventive care services to older adults in the community. Nevertheless, assessing competencies and needs is a critical initial step in organizing effective training programs, ensuring training aligns with volunteer requirements, and thus enhancing both their service attitude and intention to stay and promoting sustainable program management. In light of the above, the goals of this study were to (a) investigate the demographic characteristics of older volunteers, (b) explore the competencies and needs of older volunteers, and (c) analyze how key demographic variables influence the competencies and needs of these volunteers.

## Methods

### Design, Setting, and Participants

A comparative cross-sectional survey design was employed, and data were collected using a structured self-reporting questionnaire. The typical community care center in Taiwan has 15–25 volunteers, of which approximately 60.5% are older adults (Fu et al., 2022). Following Sudman (1976)’s recommendation of a sample size of 500–1,000 for regional surveys and assuming 9–15 older volunteers in each center, 73 community care centers were randomly selected as study sites from the 468 community care centers under the jurisdiction of the Senior Citizen Services Center of Kaohsiung (Senior Citizen Services Center, Kaohsiung, Taiwan, ROC, 2023). The enrolled sample of 1,000 individuals achieved a statistical power of 0.99 based on G Power 3.1.9.7 software. The inclusion criteria for participation were the following: (a) age ≥65 years, (b) ability to read and write Chinese, and (c) volunteer service for at least 1 month. Volunteers not actively working during the study period or who were on long-term leave during the inclusion period were excluded.

### Ethical Considerations

This study was approved by the institutional review board of Kaohsiung Medical University Chung-Ho Memorial Hospital (KMUHIRB-E(I)-20220056). The researchers conducted in-person sessions with the participants, explaining the study’s purpose and guaranteeing the anonymity of their responses. Before their involvement in the study, all of the participants provided signed informed consent. As a token of appreciation, participants received a gift valued at approximately US$3–4 upon completion of the questionnaire.

### Data Collection

The study data were collected between March and August 2024. The two-part research instrument included a demographics datasheet and the Older Volunteer Competency Scale (OVCS; Chen et al., 2024). Demographic information collected included age, gender, volunteering experience, marital status, educational level, religion, living situation, and chronic disease status. The OVCS comprises 30 items across three dimensions, including volunteer service awareness (9 items), volunteer service skills (8 items), and volunteer interpersonal interaction (13 items). The volunteer service awareness dimension is designed to investigate understanding of and insights into volunteer work; the volunteer service skills dimension is designed to investigate the operational skills required for volunteer work; and the volunteer interpersonal interaction dimension is designed to investigate the interpersonal skills that facilitate interpersonal communication during volunteer work. Respondents rate each item on a “light bulb” scale of 0–10, with 0 representing the dimmest bulb (i.e., least need of improvement) and 10 representing the brightest bulb (i.e., highest need of improvement). The use of light bulb brightness rather than numbers makes items easier for older volunteers to score accurately.

The OVCS has a high level of internal consistency, with the overall scale and its three subscales earning Cronbach’s alpha values of .98, .96, .94, and .98, respectively. Confirmatory factor analysis confirmed the scale has excellent fit, with χ² = 2.06, goodness of fit index (GFI) = .93, root mean square error of approximation (RMSEA) = .05, adjusted goodness of fit index (AGFI) = .92, normed fit index (NFI) = .96, comparative fit index (CFI) = .98, and incremental fit index (IFI) = .98 (Chen et al., 2024).

To maintain consistency during the data collection process, all of the researchers had backgrounds in gerontology and received 3 hr of data collection training from the principal investigator. The returned questionnaires were manually inspected for completeness before the data were entered into a database. Also, all input data were independently cross-examined by two researchers to ensure accuracy.

### Data Analysis

Data archiving and analysis were performed using IBM SPSS Statistics Version 25.0 (IBM Corp., Armonk, NY, USA). Descriptive statistics, including mean, standard deviation, and percentage, were used to present demographic information. Differences among demographic variables were analyzed using one-way analysis of variance with the level of significance set at *p* < .05.

## Results

### Demographic Characteristics

The participants (*N* = 1,000) were aged 65–88 years with a mean age of 71.79 years (*SD* = 4.93). The largest age group was 65–74 years (*n* = 739; 73.9%). The majority of the participants were female (*n* = 808; 80.8%), had volunteered for more than 5 years (*n* = 680; 68%), were married (*n* = 642; 64.2%), had at least a senior high school level of education (*n* = 519; 51.9%), and were not living alone (*n* = 852; 85.2%). Most had at least one chronic disease (*n* = 569; 56.9%), with hypertension (*n* = 400; 40.0%), diabetes (*n* = 154; 15.4%), and arthritis (*n* = 89; 8.9%) the most prevalent. In addition, nearly half of the participants identified as followers of Buddhism (*n* = 475; 47.5%). Further details are provided in Table 1.

**Table 1:** Participant Demographic Characteristics (N=1,000)

Characteristic	*n* (%)
**Age (years, *M* and *SD*)**	71.79 (4.93)
65–74	739 (73.9)
75–84	241 (24.1)
≥85	20 (2.0)
**Gender**	
Male	192 (19.2)
Female	808 (80.8)
**Volunteering experience (months, *M* and *SD*)**	136.01 (102.69)
≤12	47 (4.7)
13–60	273 (27.3)
≥61	680 (68.0)
**Marital status**	
Widowed or single	358 (35.8)
Married	642 (64.2)
**Educational level**	
Elementary school or lower	327 (32.7)
Junior high school	154 (15.4)
Senior high school or higher	519 (51.9)
**Religion**	
None	100 (10.0)
Buddhism	475 (47.5)
Taoism	315 (31.5)
Protestantism	100 (10.0)
Catholicism	10 (1.0)
**Living status**	
Living alone	148 (14.8)
Not living alone	852 (85.2)
**Chronic disease (*M* and *SD)***	0.83 (0.91)
No	431 (43.1)
Yes	569 (56.9)
Hypertension	400 (40.0)
Diabetes	154 (15.4)
Arthritis	89 (8.9)
Others	357 (35.7)

### Competencies and Needs of Older Volunteers

The mean OVCS score was 3.91±2.66. Among the three dimensions, volunteer service skills earned the highest score (4.49±2.76) and volunteer interpersonal interaction earned the lowest (3.54±2.86). In the volunteer service awareness dimension, the five highest scoring items and their scores, in descending order, were the following: signs of mental disorder (4.62±3.23), signs of physiological disorder (4.47±3.21), signs of acute abnormality (4.44±3.27), contagious disease prevention (4.32±3.41), and health promotion (3.96±3.41). In the volunteer service skills dimension, the top five scoring items and their scores, in descending order, were the following: designing dynamic activities (5.09±3.29), consultation and referral (4.98±3.20), designing static activities (4.96±3.32), leading dynamic activities (4.86±3.28), and leading static activities (4.72±3.29). In the volunteer interpersonal interaction dimension, the top five scoring items and their scores, in descending order, were the following: problem reporting and handling (3.96±3.11), seeking help (3.86±3.20), teamwork and conflict resolution (3.86±3.13), self-security (3.80±3.22), and observation and expressing care (3.56±3.16). Further details are provided in Table 2.

**Table 2:** Competencies and Needs

Competency/Need	*M* (*SD*)	Order
Volunteer service awareness	3.91 (2.85)	
1. Volunteer responsibilities	3.34 (3.35)	8
2. Service attitude	3.19 (3.37)	9
3. Boundary of volunteer services	3.41 (3.20)	7
4. Signs of physiological disorder	4.47 (3.21)	2
5. Signs of mental disorder	4.62 (3.23)	1
6. Signs of acute abnormality	4.44 (3.27)	3
7. Contagious disease prevention	4.32 (3.41)	4
8. Health promotion	3.96 (3.41)	5
9. Meaning of life	3.47 (3.38)	6
Volunteer service skills	4.49 (2.76)	
10. Maintaining environmental sanitation	3.22 (3.20)	8
11. Measuring and interpreting blood pressure and body temperature	3.43 (3.31)	7
12. Designing dynamic activities	5.09 (3.29)	1
13. Designing static activities	4.96 (3.32)	3
14. Leading dynamic activities	4.86 (3.28)	4
15. Leading static activities	4.72 (3.29)	5
16. Health education	4.67 (3.20)	6
17. Consultation and referral	4.98 (3.20)	2
Volunteer interpersonal interaction	3.54 (2.86)	
18. Observation and expressing care	3.56 (3.16)	4
19. Attentive listening	3.42 (3.13)	8
20. Empathy	3.46 (3.20)	6
21. Communication and interaction	3.50 (3.18)	5
22. Problem reporting and handling	3.96 (3.11)	1
23. Seeking help	3.86 (3.20)	2
24. Self-security	3.80 (3.22)	3
25. Teamwork and conflict resolution	3.86 (3.13)	2
26. Provision of timely support	3.45 (3.15)	7
27. Learning and growing as a team	3.45 (3.14)	7
28. Experience and feedback sharing	3.39 (3.13)	9
29. Mutual trust	3.11 (3.14)	11
30. Tolerance for different opinions	3.22 (3.25)	10
Overall competencies and needs	3.91 (2.66)	

### Influence of Demographic Variables on Competencies and Needs

Age, volunteering experience, and educational level were all shown to significantly influence overall competencies and needs (*F* = 4.61, *p* = .010, *F* = 3.54, *p* = .029, and *F* = 16.51, *p* < .001, respectively). Participants aged ≥85 years had more needs than those aged 75–84 years (*p* = .030); those with <1 year of volunteering experience had more needs than those with 5 years or more (*p* = .031); and those with an elementary school or lower level of education had more needs than those with a senior high school education or higher level (*p* < .001).

Age and educational level were shown to significantly influence volunteer service awareness needs (*F* = 5.65, *p* = .004 and *F* = 14.06, *p* < .001, respectively). Participants aged 65–74 years and ≥85 years had more needs than those aged 75–84 years (*p* = .030 and .031, respectively). Also, participants with an elementary school education or lower and a junior high school education had more needs than those with a senior high school education or higher (*p* < .001 and .023, respectively).

With regard to volunteer service skill needs, although a significant difference was initially found for age (*F* = 3.56, *p* = .029), further comparison revealed no significant difference between the three age groups. Volunteering experience and educational level were shown to significantly influence volunteer service skill needs (*F* = 4.23, *p* = .015 and *F* = 19.24, *p* < .001, respectively). Participants with <1 year of volunteering experience had more needs than those who had served for either 1–5 years or 5 years or more (*p* = .025 and .016, respectively), and those with an elementary school education or lower had more needs than those with a junior high school or senior high school or higher education (*p* = .020 and <.001, respectively).

With regard to volunteer interpersonal interaction needs, although a significant difference was initially found for age (*F* = 3.53, *p* = .030), further comparison revealed no significant difference between the three age groups. Volunteering experience and educational level were shown to significantly influence this needs category (*F* = 3.72, *p* = .025 and *F* = 12.67, *p* < .001). Participants with <1 year of volunteering experience had more needs than those with 5 years or more (*p* = .042), and those with an elementary school education or lower had more needs than those with senior high school education or higher (*p* < .001). Further details are provided in Table 3.

**Table 3:** Relationship Between Demographic Characteristics and Needs (*N*=1,000)

Variable	Overall Competencies and Needs			Volunteer Service Awareness			Volunteer Service Skills			Volunteer Interpersonal Interaction		
	*M*±*SD*	*F*	*p*	*M*±*SD*	*F*	*p*	*M*±*SD*	*F*	*p*	*M*±*SD*	*F*	*p*
Age (year)		4.61	.010^*^		5.65	.004^**^		3.56	.029^*^		3.53	.030^*^
① 65–74	3.98±2.66			4.02±2.87			4.55±2.72			3.60±2.87		
② 75–84	3.57±2.61			3.47±2.70			4.21±2.85			3.25±2.78		
③ ≥85	5.21±2.76			5.21±3.06			5.77±3.10			4.86±3.31		
Scheffe’s test		③ > ②	.030^*^		① > ②	.030^*^						
					③ > ②	.031^*^						
Gender		0.52	.470		0.87	.351		0.12	.725		1.25	.264
Male	4.03±2.84			4.08±2.92			4.43±2.80			3.75±3.07		
Female	3.88±2.62			3.87±2.83			4.51±2.76			3.49±2.81		
Volunteering experience (mo)		3.54	.029^*^		2.13	.119		4.23	.015^*^		3.72	.025^*^
① ≤12	4.87±2.42			4.74±2.52			5.63±2.48			4.50±2.90		
②13-60	3.95±2.71			3.90±2.82			4.44±2.73			3.69±2.91		
③ ≥61	3.82±2.65			3.86±2.87			4.43±2.78			3.41±2.83		
Scheffe’s test		① > ③	.031^*^					① > ②	.025^*^		① > ③	.042^*^
								① > ③	.016^*^			
Marital status		1.36	.244		1.49	.223		3.79	.052		0.25	.615
Unmarried	4.04±2.72			4.06±2.95			4.72±2.83			3.60±2.93		
Married	3.83±2.63			3.83±2.79			4.36±2.72			3.51±2.83		
Educational level		16.51	<.001^***^		14.06	<.001^***^		19.24	<.001^***^		12.67	<.001^***^
① Elementary school or lower	4.53±2.84			4.49±2.98			5.22±3.03			4.13±3.09		
② Junior high school	4.03±2.69			4.18±2.91			4.48±2.70			3.66±2.89		
③ Senior high school or higher	3.47±2.45			3.47±2.67			4.03±2.50			3.13±2.64		
Scheffe’s test		① > ③	<.001*		① > ③	<.001^***^		① > ②	.020^*^		① > ③	<.001^***^
					② > ③	.023^*^		① > ③	<.001^***^			
Living status		0.18	.675		0.00	.967		0.11	.740		0.53	.465
Living alone	3.99±2.68			3.90±2.92			4.56±2.84			3.70±2.89		
Not living alone	3.89±2.66			3.91±2.84			4.48±2.75			3.51±2.86		
Chronic disease		2.41	.121		1.86	.173		1.78	.183		2.57	.109
Yes	4.02±2.71			4.02±2.87			4.59±2.80			3.67±2.91		
No	3.76±2.60			3.77±2.82			4.36±2.72			3.37±2.80		

*p < .05. **p < .01. ***p < .001.

## Discussion

A majority of the participants were aged 65–74 and female, which is consistent with the findings of previous studies (Fu et al., 2022; Ministry of Health and Welfare, Taiwan, ROC, 2022). According to survey data from the Department of Household Registration, Ministry of Interior, Taiwan, ROC in 2024, 54.8% of volunteers aged 65 and above were women, and 45.2% were men. The predominance of women in this role may be attributable to their longer life expectancy than men (Lin et al., 2018; United Nations, 2022) and the influence of traditional gender roles. Women often dedicate significant time and energy to family responsibilities during their younger years and have more opportunity to pursue their desire for greater participation in society after retirement. Furthermore, the majority of participants in this study resided with their family, friends, or spouse, which is consistent with previous studies, which reported on the positive influence of support and encouragement from significant others on motivation to engage, and continue engaging, in volunteer work (Fang et al., 2017; Jongenelis et al., 2020; Lin et al., 2018).

The volunteer service skills dimension earned the highest mean score of the three dimensions. This may be attributable to the long-term practical experience required to master most volunteer service skills; aligns with the result of Fu et al. (2022); and is further supported by the higher needs level observed among participants with less volunteering experience. By contrast, and consistent with the findings of the previous study (White et al., 2020), the volunteer interpersonal interaction dimension earned the lowest mean score, possibly indicative of the skills needed in this dimension being learnable in daily life. The three top-scoring items, namely designing dynamic activities, consultation and referral, and designing static activities, are all in the volunteer service skills dimension. Among these, designing dynamic activities earned the highest score, indicating community care centers frequently organize diverse activities aimed at mitigating frailty among community-dwelling older adults, and thus, volunteers perceive a need to enhance their competency in this area. This finding aligns with that of Fu et al. (2022). In the volunteer service awareness dimension, the three top-scoring items were, in descending order, signs of mental disorder, signs of physiological disorder, and signs of acute abnormality. This indicates that, as first-line community care providers, volunteers often provide consultations on health problems in which they have a personal interest, thereby motivating them to learn relevant information. Recognizing signs of mental disorder, the most challenging of the three, typically requires a medical background or proper training. Although the high level of need for this competency may be attributable to the difficulty in identifying mental health signs, the actual reasons deserve further qualitative research.

Participant competency needs were significantly influenced by age, volunteering experience, and educational level. Overall needs were highest in the oldest age group, possibly because those in this age group were reducing their engagement in volunteer work and exposure to new information, leading to reduced confidence in related competencies and greater perceived needs. In the volunteer service awareness dimension, the needs of both the youngest and oldest age groups were higher than the middle-age group. The participants in the youngest age group were new to volunteer work and thus exhibited considerable interest in relevant knowledge, as reflected in their heightened volunteer service awareness needs. In terms of volunteering experience, needs were most pronounced among the participants with the least volunteering experience, particularly in the volunteer service skills dimension, possibly because most of these participants lacked experience designing and leading activities. By contrast, the difference between participants with more than 1 year of experience and more than 5 years of experience was statistically non-significant, suggesting that the development of volunteer service skills requires more than 1 year.

In terms of educational level, needs were higher among participants with lower educational levels, especially among those with an elementary school education or less. This may be attributable to a lower self-perception of competency, leading to higher needs. Previous studies have highlighted educational level and duration of volunteering experience as predictors of self-perceived competency and training needs that impact positively on competency development (Rizany et al., 2018; Teoh et al., 2020).

No significant association was found in this study between competency needs and living arrangements. This finding contrasts with reports in previous studies that individuals who live with others generally exhibit greater motivation to participate in volunteer work (Fang et al., 2017; Lin et al., 2018). One possible explanation for this is that individuals living alone may exhibit greater independence, reflecting greater autonomy and serving as a symbol of active aging. Moreover, those who live alone tend to have better physical and mental health than those who live with others (Fu et al., 2022; Li et al., 2009). The findings of this study suggest that older adults engaged in volunteer work at community care centers tend to have similar competency profiles regardless of residential situation. However, research on the connection between living arrangements and competency level remains limited. Independence may play a role in this relationship, warranting further empirical investigation in the future.

### Limitations

Because of the cross-sectional design used and the use of self-reported data, the results of this study are based solely on participant responses, which limits their utility for in-depth investigations or establishing causal relationships. Moreover, because the participants comprised older volunteers serving in community care centers in southern Taiwan, the results may not be generalizable to volunteers in other regions or age groups. Finally, because standard deviations were not considered and only mean scores were used in the analysis, caution must be exercised in interpreting the research results.

### Conclusions

The 1,000 participants in this study were all older volunteers working at community care centers. Age, volunteering experience, and educational level were each found to exhibit significant influence over the overall level and dimensions of needs. The oldest age group and participants with the least experience and lowest educational level reported the highest requirements in terms of overall competencies and needs. In terms of service awareness, both the youngest and oldest groups, along with participants with an elementary school or lower and junior high school education, showed higher needs; in terms of service skills, those with the least experience and with the lowest educational level showed higher needs; and, in terms of interpersonal interaction, those with the least experience and lowest educational level showed higher needs.

The following recommendations are proposed based on the research findings: (a) Regular workshops should be organized to enhance service awareness in volunteers. Workshop content must consider the age, educational level, and physical and mental conditions of older volunteers. (b) Community care centers should provide more opportunities to practice designing and leading both dynamic and static activities to enhance the service skills of their volunteers. (c) To improve consultation and referral competencies, monthly meetings should be held with volunteers, focused on how to handle special or problem cases. (d) Given that the division of labor among volunteers can be highly task-specific, future studies should group volunteers by specific tasks to increase the specificity of research results. (e) As the assessment of competencies and needs is an initial step toward organizing training programs, course planners should survey their volunteers using the OVCS to ensure program content matches volunteer needs. (f) Community care centers are encouraged to review the competencies of their volunteers routinely to facilitate appropriate task assignment. (g) Due to the consistently reported higher number of female volunteers than males, community care centers should organize appropriate activities to attract more male volunteers. Also, community care centers should assign tasks to their volunteers based on their competencies to enhance service satisfaction. (h) In future research, training programs should be designed based on the competencies and needs of older volunteers, and the impact of these programs on the long-term engagement and satisfaction of older volunteers should be investigated.
